# The Effect of Enantiomer Elution Order on the Determination of Minor Enantiomeric Impurity in Ketoprofen and Enantiomeric Purity Evaluation of Commercially Available Dexketoprofen Formulations

**DOI:** 10.3390/molecules25245865

**Published:** 2020-12-11

**Authors:** Kenan Can Tok, Mehmet Gumustas, Giorgi Jibuti, Halit Sinan Suzen, Sibel A. Ozkan, Bezhan Chankvetadze

**Affiliations:** 1 Department of Forensic Toxicology, Institute of Forensic Sciences, Ankara University, Ankara 06590, Turkey; kctok@ankara.edu.tr (K.C.T.); mgumustas@ankara.edu.tr (M.G.); sinan.suzen@ankara.edu.tr (H.S.S.); 2 Institute of Physical and Analytical Chemistry, School of Exact and Natural Sciences, Tbilisi State University, Chavchavadze Ave 3, 0179 Tbilisi, Georgia; giorgi.jibuti@tsu.ge; 3 Department of Pharmaceutical Toxicology, Faculty of Pharmacy, Ankara University, Ankara 06560, Turkey; 4 Department of Analytical Chemistry, Faculty of Pharmacy, Ankara University, Ankara 06560, Turkey; ozkan@pharmacy.ankara.edu.tr

**Keywords:** enantiomeric impurity determination, amylose tris(3-chloro-5-methylphenylcarbamate), enantiomer elution order reversal, method validation, dexketoprofen

## Abstract

In a recent study, opposite enantiomer elution order was observed for ketoprofen enantiomers on two amylose-phenylcarbamate-based chiral columns with the same chemical composition of the chiral selector but in one case with coated while in the other with an immobilized chiral selector. In the present study, the influence of this uncommon effect on method validation parameters for the determination of minor enantiomeric impurity in dexketoprofen was studied. The validated methods with two alternative elution orders for enantiomers were applied for the evaluation of enantiomeric impurity in six marketed dexketoprofen formulations from various vendors. In most of these formulations except one the content of enantiomeric impurity exceeded 0.1% (*w*/*w*).

## 1. Introduction

The difference in pharmacokinetic, pharmacodynamic, metabolic, and toxic properties between the enantiomers of chiral drugs and other biologically active compounds is well known [1,2,3]. On one side this fact and on the other, the rapid development of state-of-the-art technologies for the manufacturing [4,5,6,7,8] and analysis [8,9,10] of single enantiomers of chiral compounds enabled the commercialization of enantiomerically pure novel chiral drugs, as well as redevelopment of many well-known chiral drugs previously used as racemates in enantiomerically pure form (so-called chiral switch) [11,12,13]. Currently, some chiral drugs are offered in both racemic as well as in enantiomerically pure formulations. The latter are more expensive claiming significant therapeutic advantages such as higher therapeutic index, lower toxicity, simplification of the dose–response relationship, and more selective pharmacodynamic profile [8,11,12,13].

**Figure 1:**
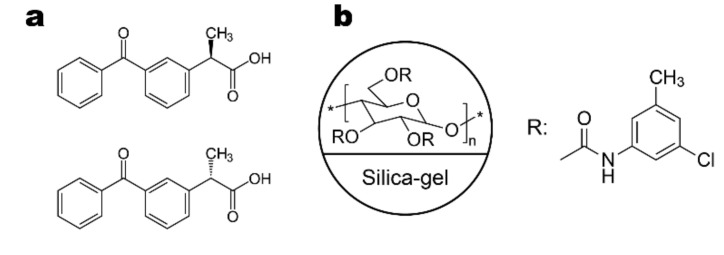
Structure of ketoprofen (**a**) and chiral selector (**b**).

The evaluation of enantiomeric purity of single-enantiomer chiral drug formulations is quite a hot topic nowadays. It has been considered for many years that eluting the minor impurity in front of the major component offers certain advantages for more precise quantification of the impurity [10,14,15,16,17]. Choosing a more favorable enantiomer elution order (EEO) in chromatographic separations is easily possible with the chiral selectors which are available in both stereochemical configurations. To this group belong quite many brush-type chiral selectors [18], quinine- and quinidine-based ion exchangers [19], and a few other groups of rarely used chiral selectors. Many widely used chiral selectors, such as polysaccharide derivatives [9,10], cyclodextrin derivatives [20], glycopeptide antibiotics [21], and proteins [22] are available in nature only in single stereochemical configuration. Thus, the easy reversal of EEO by alternative use of two chiral columns with the opposite stereochemical configuration of the chiral selectors is not an option for these chiral columns. Despite the same stereochemical configuration of the basic chiral units in polysaccharide-based chiral columns, the reversal of EEO can be achieved by changing the chemistry of a chiral selector [10,14,23,24,25], mobile phase composition [1,10,23,24,25,26,27,28,29,30,31,32,33], mobile phase polar modifier [10,23,24,25,26,27,28,29,30,31,32,33], mobile phase additive and/or its concentration [10,17,33,34,35], or separation temperature [1,10,33,36]. In order to study the effect of EEO on method validation parameters for the determination of a minor enantiomeric impurity (*R*-ketoprofen) in a single-enantiomer drug formulation (*S*-ketoprofen) (Figure 1a), it seems desirable to achieve a reversal in EEO by some minor modification of the column chemistry. In this study, two chiral columns containing amylose tris(3-chloro-5-methylphenylcarbamate) as a chiral selector (Figure 1b) were used for developing HPLC methods for enantiomeric impurity determination in several formulations of dexketoprofen (Figure 1a). The methods were compared to each other based on method validation parameters and then applied to enantiomeric impurity determination in commercially available injectable dexketoprofen solutions.

## 2. Results

### 2.1. Method Development for the Separation of Ketoprofen Enantiomers on Lux i-Amylose-3 Column and Its Analogue with a Coated Chiral Selector

**Figure 2:**
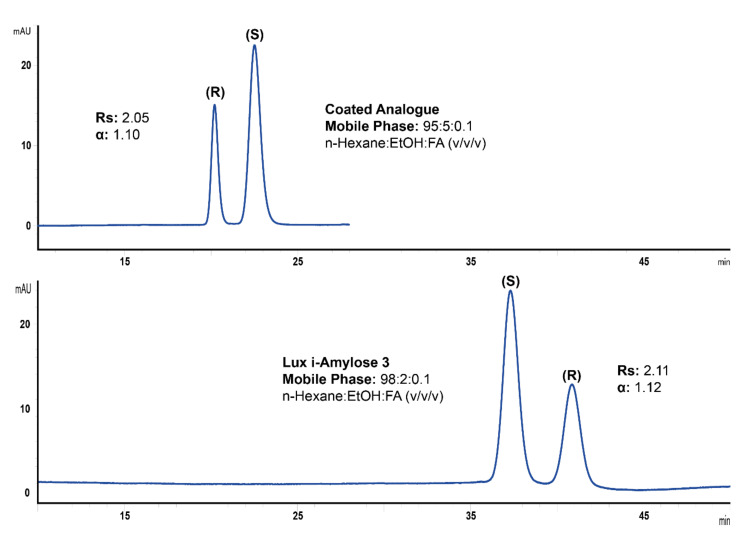
Separation of *R*- and *S*-ketoprofen mixture (1/2 ratio, *w*/*w*) on amylose tris(3-chloro-5-methylphenylcarbamate)-based chiral columns (250 × 4.6 mm, 5 µm) with coated (**a**) and immobilized (**b**) chiral selector. Separation temperature was 35 °C, flow rate was 2 mL/min, detection was performed at 254 nm with the mobile phase composition of n-hexane:ethanol:formic acid, 95:5:0.1 (*v*/*v*/*v*) for coated (**a**) and n-hexane:ethanol:formic acid, 98:2:0.1 (*v*/*v*/*v*) for immobilized (**b**) chiral selector.

As mentioned above, the major goal of this study was to evaluate the effect of EEO on method validation parameters while keeping other factors minimal. First of all, in our opinion, keeping separation factors as close as possible to each other on both columns is necessary in order to avoid any bias on method validation parameters. Previously reported reversal of the EEO for ketoprofen on the Lux i-Amylose-3 column and its analogue with a coated chiral selector [1] was confirmed in the present study (Figure 2). However, some adjustment of the mobile phase was necessary in order to obtain comparable separation factors (α) on both columns. In addition, a separation factor in the range of 1.1–1.2 is commonly observed in successful HPLC separations of enantiomers with acceptable analyte retention and thus, seems to be representative of the most common cases in practice [17,23,24]. The separation of ketoprofen enantiomers, more or less meeting the mentioned requirements, on both studied columns are shown in Figure 2.

As one can see from this figure, a lower percentage of a polar mobile phase modifier was required with Lux i-Amylose-3 with an immobilized chiral selector compared to its coated analogue. As a result, the analysis time on the former column was almost twice as long compared to the latter column. Long analysis times are undesirable, and also lead to increased mobile phase consumption as well as may cause unfavorable separation efficiency and peak shapes. The system suitability tests shown in Table 1 for both columns indicate that the peak symmetry was slightly better on the column with an immobilized chiral selector.

**Figure 3:**
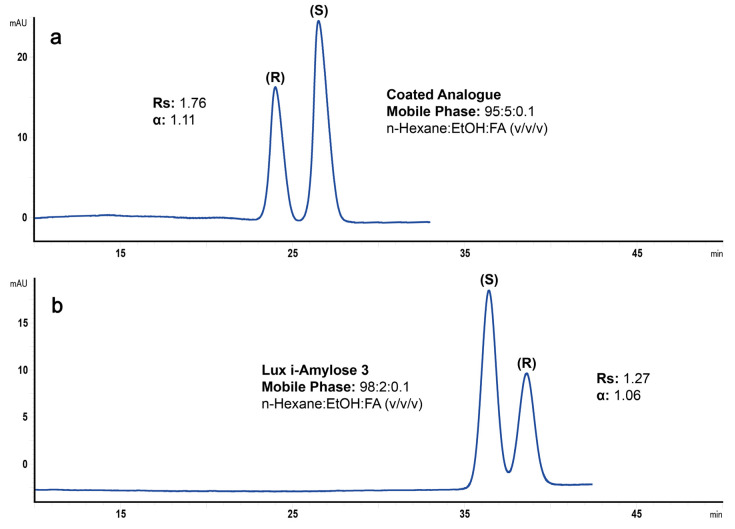
Separation of *R*- and *S*-ketoprofen mixture (1/2 ratio, *w*/*w*) on amylose tris(3-chloro-5-methylphenylcarbamate)-based chiral columns (250 × 4.6 mm, 5 µm) with coated (**a**) and immobilized (**b**) chiral selectors. Separation temperature was 25 °C, flow rate was 2 mL/min, detection was performed at 254 nm with the mobile phase composition of n-hexane:ethanol:formic acid, 95:5:0.1 (*v*/*v*/*v*) for the coated (**a**) and n-hexane:ethanol:formic acid, 98:2:0.1 (*v*/*v*/*v*) for the immobilized (**b**) chiral selector.

In addition to the mobile phase composition, the separation temperature had to also be optimized for obtaining comparable system suitability test results on both columns. The separation of ketoprofen enantiomers on both columns at 25 °C are shown in Figure 3.

Clearly, the separation factor on the column with the immobilized chiral selector is lower while the analysis time is longer compared to the column with the coated chiral selector. Considering that the retention of ketoprofen enantiomers on this column is enthalpy-controlled while their separation above 0 °C is mostly entropy-controlled [1], the increase in separation temperature up to 35 °C was undertaken in order to improve separation selectivity. The same increase in temperature on the column with the coated chiral selector led to a decrease in analysis time as well as to a slight decrease in the separation factor (the separation of ketoprofen enantiomers is enthalpy-controlled up to about 45 °C on this column [1]). This enabled us to bring the separation factor closer to each other and thus, avoid any bias on the method validation parameters.

### 2.2. Method Validation Results and Application to Racemic Ketoprofen Formulation

Currently, ketoprofen formulations available on the market contain either racemic active pharmaceutical ingredient (API) or its *S*-enantiomer (dexketoprofen). Therefore, two validated methods were developed: one for the determination of the content of the API in formulations of racemic ketoprofen, as well as dexketoprofen, and the other one for the evaluation of enantiomeric purity of API in commercially available dexketoprofen formulations. The method validation parameters are summarized in Table 2, and calibration curves and regression equations are shown in the Supplementary Figure S1.

**Figure 4:**
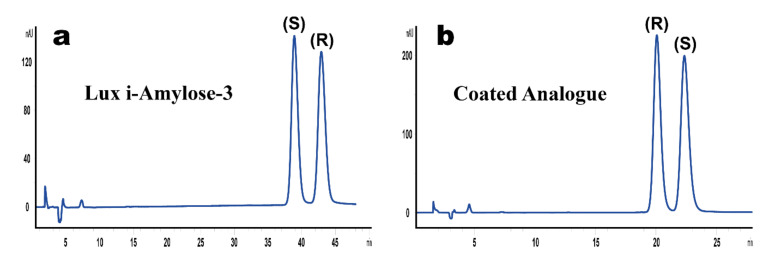
The results of chromatographic analysis of racemic ketoprofen 50 mg/mL injectable solution with both methods. Separation temperature was 35 °C, flow rate was 2 mL/min, detection was performed at 254 nm with the mobile phase composition of n-hexane:ethanol:formic acid, 95:5:0.1 (*v*/*v*/*v*) for coated (**a**) and n-hexane:ethanol:formic acid, 98:2:0.1 (*v*/*v*/*v*) for immobilized (**b**) chiral selector.

The result of analysis of commercially available liquid formulation of ketoprofen for intramuscular injection with the stated content of 50 mg/mL racemic ketoprofen is shown in Figure 4 and Table 3. As one can see from this figure, the results obtained for racemic formulation on both columns are comparable and from the viewpoint of method validation parameters, almost no preference can be given to any method (i.e., preference for elution order of enantiomers).

### 2.3. Determination of Minor Enantiomeric Impurity and Method Applicability to Dexketoprofen Formulations

Calibration line for determination of *R*-ketoprofen minor impurity in *S*-ketoprofen formulations was constructed in the range of 0.01–2.50% (*w*/*w*). The calibration line and regression equation are shown in Figure S2. Recovery experiments were performed on three commercially available formulations of dexketoprofen with the stated content of the active ingredient to be 25 mg/mL. The results are shown in Table 4.

**Figure 5:**
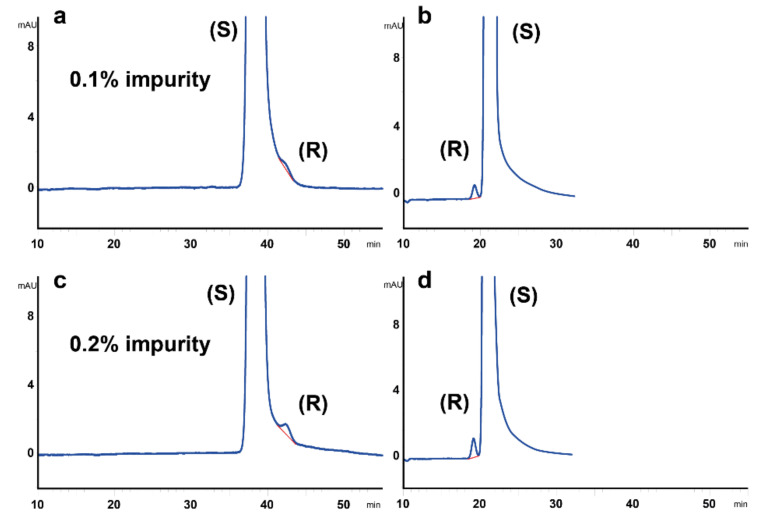
The chromatograms of two commercially available dexketoprofen 25 mg/mL injectable solutions with methods using either Lux i-Amylose-3 (250 × 4.6 mm, 5 µm) (**a**,**c**), or its coated analogue (250 × 4.6, 5 µm) (**b**,**d**). Separation temperature was 35 °C, flow rate was 2 mL/min, detection was performed at 254 nm with the mobile phase composition of n-hexane:ethanol:formic acid, 95:5:0.1 (*v*/*v*/*v*) for coated and n-hexane:ethanol:formic acid, 98:2:0.1 (*v*/*v*/*v*) for immobilized chiral selector.

In contrast to the case with racemic formulation, the advantage of the method in which the minor enantiomer elutes in front of the major one becomes obvious when the method is aimed at determining the minor enantiomeric impurity in dexketoprofen formulation. Thus, the limits of detection (LOD) and limits of quantification (LOQ) of the minor enantiomeric impurity are lower when its peak elutes first (Table 2). This can be easily seen also from the chromatograms shown in Figure 5 for two different commercially available formulations of dexketoprofen.

Thus, the earlier reached conclusion regarding the advantage of eluting the minor impurity before the major component was clearly observed also in the present study. The results for analyte recovery and method accuracy summarized in Table 4 and Table 5 also demonstrate the advantages of eluting the minor impurity in front of the major peak. The quite surprising result of this study was that 5 of 6 studied enantiomerically pure formulations of dexketoprofen contained the impurity of *R*-ketoprofen higher than 0.1% (*w*/*w*) level.

## 3. Materials and Methods

### 3.1. Materials

The chiral test compounds, racemic ketoprofen and *S*-(+) ketoprofen (dexketoprofen) were supplied from Nobel Ilac (Duzce, Istanbul, Turkey), and its *R*-(-)-enantiomer was commercially available from Sigma-Aldrich (St. Louis, MO, USA). The structure of the studied analyte is shown in Figure 1a. Commercially available racemic injectable ketoprofen formulation with 50 mg/mL stated concentration of racemic ketoprofen (2 mL ampules) was sourced from a pharmacy shop in Tbilisi, Georgia, and dexketoprofen injectable formulations with 25 mg/mL (2 mL) declared concentration of active ingredient from six different pharmaceutical companies, were acquired in pharmacy shops in Ankara, Turkey and Tbilisi, Georgia. HPLC-grade n-hexane and ethanol as well as chemical-grade formic acid, were supplied by Karl Roth (Karlsruhe, Germany). Chiral column Lux i-Amylose-3 was provided by Phenomenex Inc. (Torrance, CA, USA). The coated version of the Lux i-Amylose-3 column was prepared in our laboratory based on the method described earlier [37]. The structure of the chiral selector is shown in Figure 1b. Both columns were of 250 × 4.6 mm dimensions packed with silica particles of 5 µm nominal particle size.

### 3.2. Instrument

An Agilent 1200 HPLC instrument (Agilent Technologies, Palo Alto, CA, USA) equipped with a G1367C HiP ALS-SL autosampler, G1316B TCC-SL temperature controller, G1311A quaternary pump, G1314D VWD variable wavelength detector, including with the Chemstation software (version B.03.02-SR2) was used for instrument control, data acquisition, and data processing. HPLC separations were performed at 308 K for the coated and covalently immobilized columns, respectively at 2.00 mL/min mobile phase flow rate and 10 µL injection volume if not mentioned otherwise. All mobile phases contained 0.1% formic acid (*v*/*v*). UV detection was performed at 254 nm. The absolute configuration of enantiomers was assigned based on a spiking experiment with enantiomerically pure standards.

### 3.3. Method Validation

Method validation was performed according to International Council for Harmonisation Guidelines [38]. In particular, the system suitability test parameters of the method, linearity range, limit of detection, limit of quantification, precision, and accuracy were determined as follows: for determining the linearity of the developed method, stock solutions of *S*-ketoprofen (dexketoprofen) and *R*-ketoprofen (minor impurity) were prepared by dissolving 2 mg/mL and 0.1 mg/mL, respectively in methanol. If required, the aliquots were further diluted with methanol.

System suitability for the proposed method was evaluated according to the official criteria including capacity factor (k), resolution (Rs), theoretical plates number (N), retention time (t), tailing factor (T_f_), symmetry, and separation factor (α).

Calibration curve for determination of *R*-ketoprofen minor impurity in *S*-ketoprofen was constructed in the range of 0.01–2.50% (*w*/*w*). The vials containing the standards and the samples for the injections were placed into the autosampler at 4 °C. The calibration lines for the determination of content of ketoprofen enantiomers in its racemic formulation was constructed in the co-ordinates content vs. peak area. The calibration lines for determination of content of enantiomeric impurity of *R*-ketoprofen in dexketoprofen formulations were constructed by plotting the percentage of *R*-ketoprofen (*w*/*w*) vs. the ratio of the peak area of *R*-ketoprofen over the sum of the peak area of both enantiomers.

The precision of the methods was determined by analyzing three different concentrations for each enantiomer on the same day and on three consecutive days, respectively. The summarized results are reported as RSD% values.

Accuracy of the methods was examined by spiking the selected commercial formulations with the known amount of standard solution of the *R*-ketoprofen. Stock solution was prepared at 0.1 mg/mL concentration in methanol in a volumetric flask. In order to investigate the effect of excipients on the assay, recovery studies were carried out by adding a known amount of pure active substance to the commercial formulation solutions. The results were obtained from five replicate analyses.

## 4. Conclusions

In the present study, two methods were developed for the determination of minor enantiomeric impurity of *R*-ketoprofen in commercially available *S*-ketoprofen formulations (dexketoprofen). Comparison of validation parameters of the two methods with opposite elution order of enantiomers confirmed earlier conclusions about the advantages of eluting the minor enantiomer in front of the major one. In particular, the limit of detection and limit of quantification were lower and recovery and accuracy of determination were better for a minor enantiomeric impurity when it was eluting in front of the major enantiomer. Of the studied six dexketoprofen formulations on the market, five had a content of enantiomeric impurity (*R*-ketoprofen) that exceeded 0.1% (*w*/*w*).

## Figures and Tables

**Table 1:** System suitability tests on amylose tris(3-chloro-5-methylphenylcarbamate)-based columns with immobilized and coated chiral selectors.

Parameters	Lux i-Amylose-3		Coated Analogue	
	*S*-Enantiomer	*R*-Enantiomer	*R*-Enantiomer	*S*-Enantiomer
Retention time (min)	37.31	40.86	20.23	22.54
Capacity factor (k)	8.33	9.22	4.07	4.64
Theoretical plates numbers (N)	8101	8337	5666	5499
Separation factor (α)	-	1.12	-	1.10
Resolution (Rs)	-	2.11	-	2.05
Symmetry	0.94	0.92	0.85	0.85
Tailing factor (Tf)	1.06	1.00	1.16	1.17

**Table 2:** Summary of the calibration results for dexketoprofen.

Parameters	Lux i-Amylose-3		Coated Analogue	
	*S*-Enantiomer	*R*-Enantiomer	*R*-Enantiomer	*S*-Enantiomer
Linearity range (mg/mL)	0.1–100	0.01–2.5%	0.01–2.5%	0.1–100
Slope	19.72 × 10^3^	0.0011	0.0012	19.64 × 10^3^
Determination coefficient (R^2^)	0.9999	0.9997	0.9999	0.9999
Intercept	6.3 × 10^3^	8 × 10^−5^	2 × 10^−4^	4.7 × 10^3^
Limit of detection (µg/mL)	0.033	0.033	0.01	0.01
Limit of quantification (µg/mL)	0.10	0.10	0.03	0.03
Within-day precision (RSD, %; *n* = 5)	2.80	3.29	1.69	0.70
Between-day precision (RSD, %; *n* = 5)	3.13	3.65	2.59	1.12

**Table 3:** The results of chromatographic analysis of racemic ketoprofen.

	Lux i-Amylose-3		Coated Analogue	
	*S*-Enantiomer	*R*-Enantiomer	*R*-Enantiomer	*S*-Enantiomer
Labeled Amount (mg/mL)	50		50	
Found Amount (mg/mL)	23.89	23.78	24.18	23.92
Total Amount (mg/mL)	47.67		48.10	
RSD (%) *	0.46	0.59	0.12	0.13
Bias (%) *	4.46	4.91	3.28	4.32
Total Bias (%) *	4.66		3.80	

* *n* = 5.

**Table 4:** Recovery results for selected formulations.

Formulation	Lux i-Amylose-3				Coated Analogue			
	Added Amount (%)	Found Amount (%)	RSD (%) *	Recovery (%) *	Added Amount (%)	Found Amount (%)	RSD (%) *	Recovery (%) *
Formulation 1	0.5	0.493	4.20	98.51	0.5	0.509	3.70	101.80
Formulation 2	0.5	0.510	2.35	101.99	0.5	0.501	0.77	100.20
Formulation 6	0.5	0.477	3.30	95.36	0.5	0.503	2.90	100.68

* *n* = 5.

**Table 5:** Content of ketoprofen enantiomers in marketed dexketoprofen formulations.

	Dexketoprofen Formulation	F1	F2	F3	F4	F5	F6
Lux i-Amylose-3	Content of *S*-Enantiomer, mg/mL	23.44	23.74	24.64	24.43	24.73	23.42
	Content of *R*-Enantiomer, mg/mL	0.09	0.49	0.14	0.02	0.06	0.04
	Enantiomeric impurity of dexketoprofen, % (*w*/*w*)	0.34	1.95	0.57	0.07	0.24	0.16
	RSD (%) *	3.09	4.42	3.69	2.66	4.19	3.86
	Total content of ketoprofen, mg/mL	23.53	24.23	24.78	24.45	24.79	23.46
	Declared content of dexketoprofen, mg/mL	25	25	25	25	25	25
	Bias (%)	5.88	3.08	0.88	2.20	0.84	6.16
							
Coated Analogue	Content of *S*-Enantiomer, mg/mL	23.66	24.38	24.95	25.01	24.91	24.36
	Content of *R*-Enantiomer, mg/mL	0.09	0.42	0.13	0.03	0.07	0.04
	Enantiomeric impurity of dexketoprofen, % (*w*/*w*)	0.34	1.66	0.50	0.10	0.26	0.17
	RSD (%) *	0.39	0.22	0.58	1.90	2.47	3.18
	Total content of ketoprofen, mg/mL	23.75	24.80	25.08	25.04	24.98	24.40
	Stated content of dexketoprofen, mg/mL	25	25	25	25	25	25
	Bias (%) *	5.00	0.80	−0.32	−0.16	0.08	2.40

* *n* = 5.

## Supplementary Materials

Supplementary materials are available online. Figure S1a: Chromatogram and calibration curve for racemic ketoprofen by using Lux i-Amylose-3. Separation temperature was 35 °C, flow rate was 2 mL/min, detection was performed at 254 nm with the mobile phase composition of n-Hexane:ethanol:formic acid, 98:2:0.1 (*v*/*v*/*v*); Figure S1b: Chromatogram and calibration curve for racemic ketoprofen by using coated analogue of Lux i-Amylose-3. Separation temperature was 35 °C, flow rate was 2 mL/min, detection was performed at 254 nm with the mobile phase composition of n-Hexane:ethanol:formic acid, 95:5:0.1 (*v*/*v*/*v*); Figure S2: The calibration levels for the impurity: Constant amount of (*S*)-ketoprofen and the increasing amount of (*R*)-ketoprofen (bottom to top: 0.01–2.50% (*w*/*w*), *n* = 7). For experimental conditions see Section 2.1 and Section 2.2.
