# Supplementary material for: The Effect of Enantiomer Elution Order on the Determination of Minor Enantiomeric Impurity in Ketoprofen and Enantiomeric Purity Evaluation of Commercially Available Dexketoprofen Formulations
Source: Molecules. 2020 Dec 11;25(24):5865. doi: 10.3390/molecules25245865 (PMC7763306; doi:10.3390/molecules25245865)
Supplement: Supplementary file 1 [file molecules-25-05865-s001.zip › molecules-1018916-supplementary.docx]

Article

The Effect of Enantiomer Elution Order on the Determination of Minor Enantiomeric Impurity in Ketoprofen and Enantiomeric Purity Evaluation of Commercially Available Dexketoprofen Formulations

Kenan Can Tok ^1^, Mehmet Gumustas ^1^, Giorgi Jibuti ^2^, H. Sinan Suzen ^1,3^, Sibel A. Ozkan ^4^ and Bezhan Chankvetadze ^2,^*

^1^ Department of Forensic Toxicology, Institute of Forensic Sciences, Ankara University, 06590 Ankara, Turkey; [kcantok@gmail.com](mailto:kcantok@gmail.com) (K.C.T.); [mgumustas@ankara.edu.tr](mailto:mgumustas@ankara.edu.tr) (M.G.); [hssuzen@gmail.com](mailto:hssuzen@gmail.com) (H.S.S.)

^2^ Institute of Physical and Analytical Chemistry, School of Exact and Natural Sciences, Tbilisi State University, Chavchavadze Ave 3, 0179 Tbilisi, Georgia; [giorgi.jibuti@tsu.ge](mailto:giorgi.jibuti@tsu.ge) (G.J)

^3^ Department of Pharmaceutical Toxicology, Faculty of Pharmacy, Ankara University, 06560 Ankara, Turkey

^4^ Department of Analytical Chemistry, Faculty of Pharmacy, Ankara University, 06560 Ankara, Turkey; [ozkan@pharmacy.ankara.edu.tr](mailto:ozkan@pharmacy.ankara.edu.tr) (S.A.O)

***** Correspondence: [bezhan.chankvetadze@tsu.ge](mailto:bezhan.chankvetadze@tsu.ge) (B.C); Tel.: +995-595-631900


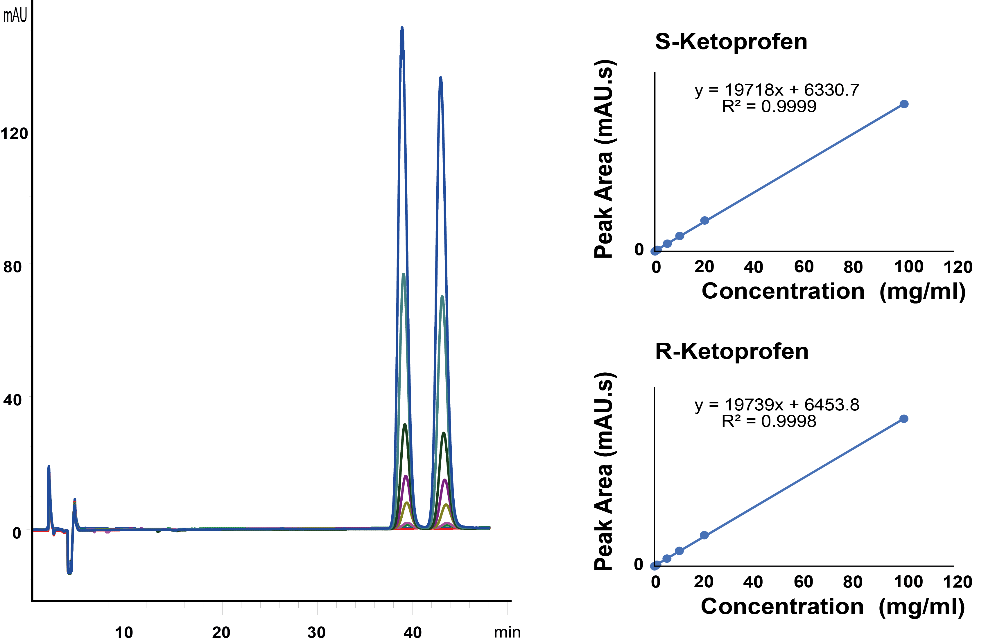


**Figure S1a.** Chromatogram and calibration curve for racemic ketoprofen by using Lux-i Amylose-3. Separation temperature was 35°C, flow rate was 2 ml/min, detection was performed at 254nm with the mobile phase composition of n-Hexane:ethanol:formic acid, 98:2:0.1 (*v/v/v*).


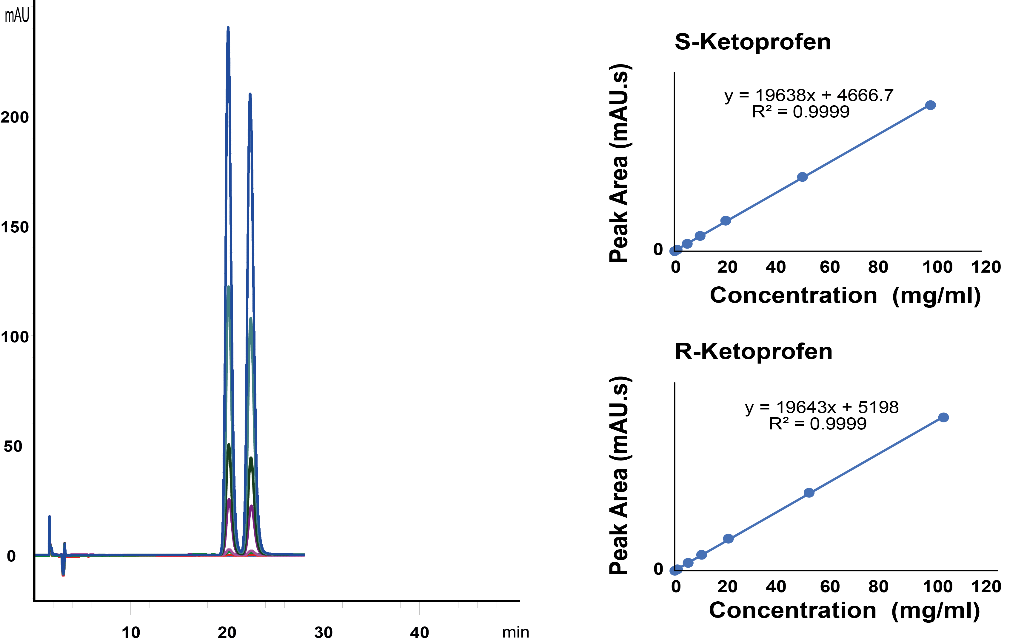


**Figure S1b.** Chromatogram and calibration curve for racemic ketoprofen by using coated analogue of Lux-i Amylose-3. Separation temperature was 35°C, flow rate was 2 ml/min, detection was performed at 254nm with the mobile phase composition of n-Hexane:ethanol:formic acid, 95:5:0.1 (*v/v/v*).


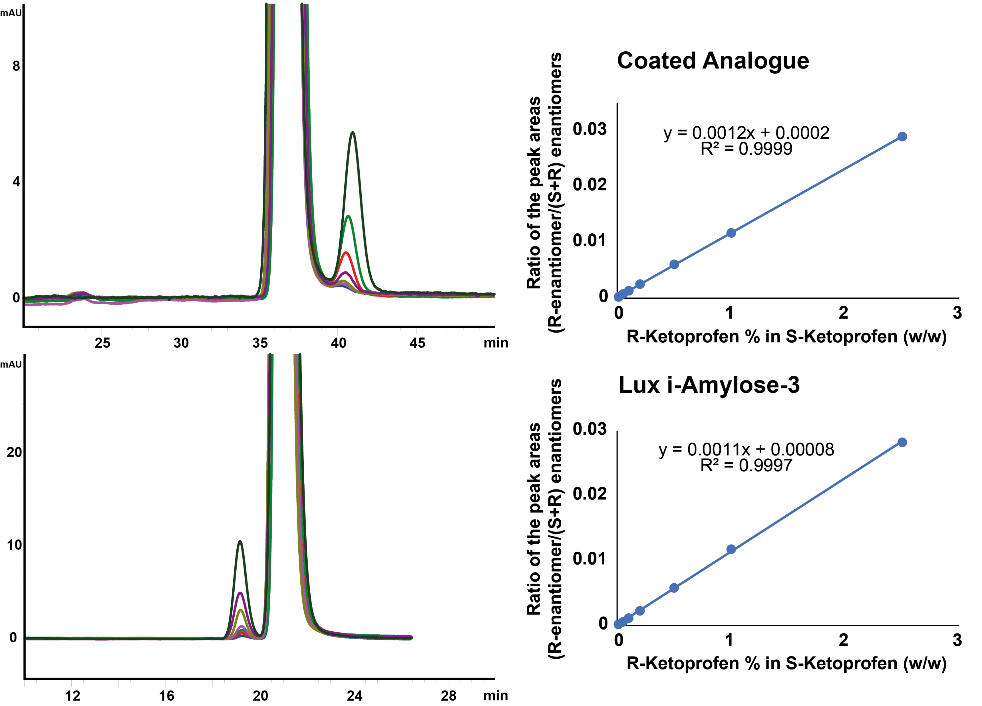


**Figure S2.** The calibration levels for the impurity: Constant amount of (*S*)-ketoprofen and the increasing amount of (*R*)-ketoprofen (bottom to top: 0.01%-2.50 % (*w/w*), n=7). For experimental conditions see subsections 2.1 and 2.2.
